# Ror2 Signaling and Its Relevance in Breast Cancer Progression

**DOI:** 10.3389/fonc.2017.00135

**Published:** 2017-06-26

**Authors:** Michaela Bayerlová, Kerstin Menck, Florian Klemm, Alexander Wolff, Tobias Pukrop, Claudia Binder, Tim Beißbarth, Annalen Bleckmann

**Affiliations:** ^1^Department of Medical Statistics, University Medical Center Göttingen, Göttingen, Germany; ^2^Department of Hematology and Medical Oncology, University Medical Center Göttingen, Göttingen, Germany; ^3^Clinic for Internal Medicine III, Hematology and Medical Oncology, University Regensburg, Regensburg, Germany

**Keywords:** Wnt signaling, Ror2, network integration, module, breast cancer, metastasis

## Abstract

Breast cancer is a heterogeneous disease and has been classified into five molecular subtypes based on gene expression profiles. Signaling processes linked to different breast cancer molecular subtypes and different clinical outcomes are still poorly understood. Aberrant regulation of Wnt signaling has been implicated in breast cancer progression. In particular Ror1/2 receptors and several other members of the non-canonical Wnt signaling pathway were associated with aggressive breast cancer behavior. However, Wnt signals are mediated *via* multiple complex pathways, and it is clinically important to determine which particular Wnt cascades, including their domains and targets, are deregulated in poor prognosis breast cancer. To investigate activation and outcome of the Ror2-dependent non-canonical Wnt signaling pathway, we overexpressed the Ror2 receptor in MCF-7 and MDA-MB231 breast cancer cells, stimulated the cells with its ligand Wnt5a, and we knocked-down Ror1 in MDA-MB231 cells. We measured the invasive capacity of perturbed cells to assess phenotypic changes, and mRNA was profiled to quantify gene expression changes. Differentially expressed genes were integrated into a literature-based non-canonical Wnt signaling network. The results were further used in the analysis of an independent dataset of breast cancer patients with metastasis-free survival annotation. Overexpression of the Ror2 receptor, stimulation with Wnt5a, as well as the combination of both perturbations enhanced invasiveness of MCF-7 cells. The expression–responsive targets of Ror2 overexpression in MCF-7 induced a Ror2/Wnt module of the non-canonical Wnt signaling pathway. These targets alter regulation of other pathways involved in cell remodeling processing and cell metabolism. Furthermore, the genes of the Ror2/Wnt module were assessed as a gene signature in patient gene expression data and showed an association with clinical outcome. In summary, results of this study indicate a role of a newly defined Ror2/Wnt module in breast cancer progression and present a link between Ror2 expression and increased cell invasiveness.

## Introduction

Breast cancer is a heterogeneous disease with respect to pathological characteristics, molecular profiles, and prognoses. Gene signatures derived from gene expression profiles proved to be useful to separate breast cancers into distinct molecular subtypes. Based on the PAM50 gene signature, five subtypes have been defined: Basal-like (Basal), ERBB2-overexpressing (Her2), luminal A (LumA), luminal B (LumB), and normal-breast-like breast cancer (1, 2). They have been associated with significant differences in clinical outcome in terms of developing distant metastasis and overall survival (3). Furthermore, these subtypes vary in activation states of multiple signaling pathways, among them the Wnt signaling pathway. Aberrant regulation of Wnt signaling has been implicated in breast cancer progression (4) and expression of a number of important Wnt pathway members has been shown to be altered in different molecular subtypes (5).

However, Wnt signals are channeled through several distinct cascades. Activation of the canonical, β-catenin-dependent Wnt pathway is characterized by the accumulation of β-catenin in the cytosol and its translocation to the nucleus. Subsequent transcription changes determine cell survival and proliferation (6). In contrast, alternative non-canonical Wnt pathways mediate β-catenin-independent signals. Multiple non-canonical Wnt ligands bind receptors, such as Ror1, Ror2, Ryk, and several members of the Frizzled receptor family, in a rather promiscuous way. Three main cascades can be distinguished: Wnt/Ror signaling, Wnt/Ca2+ signaling, and Wnt/planar cell polarity (PCP); however, these cascades are greatly intertwined (7). For example, the Wnt5a ligand can bind Ror1/Ror2 tyrosine kinase receptors, which activate Jun-N-terminal kinase (Jnk). Subsequently, this initiates transcription *via* the c-Jun transcription factor and can inhibit β-catenin-dependent Wnt signaling. Moreover, Wnt5a can also traffic signals toward the PCP cascade *via* RhoA, Rac, and Cdc42. The outcome of non-canonical Wnt signaling in general is linked to cytoskeletal rearrangements and changes in cell motility (7–10).

Several particular non-canonical pathway members have been associated with aggressive breast cancer subtypes. For instance, Wnt5a and Wnt5b were found to be overexpressed in basal-like MDA-MB-231 cells compared to less aggressive LumA MCF-7 cells and their expression levels were also elevated together with Ror1/Ror2 in breast cancer brain metastases (11). Furthermore, breast cancer patients expressing Ror1 and Ror2 have been reported to show a poor survival (12, 13). However, specific outcomes of distinct Wnt signaling pathways triggered by a particular ligand–receptor binding are still poorly understood in the context of breast cancer.

**Figure 1:**
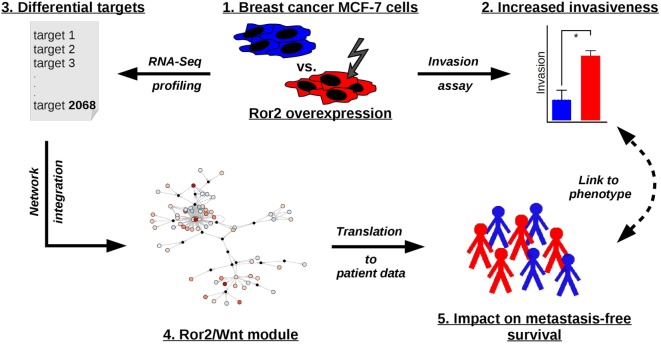
Conceptual workflow of the study. (1.) We explored activation and outcome of the non-canonical Wnt signaling by overexpression of the Ror2 receptor in the ER+ breast cancer cell line MCF-7. (2.) To explore the effect of perturbation on the phenotypic level, the invasive capacity of the cells was measured and (3.) on gene expression level the mRNA of the cell lines was profiled. (4.) The results of differential analysis were integrated with the non-canonical Wnt signaling network to identify an induced subnetwork—the Ror2/Wnt module. (5.) The Ror2/Wnt module genes were further translated into independent gene expression data of breast cancer patients in order to assess the association of non-canonical Ror2/Wnt signaling with metastasis-free survival in breast cancer.

Here, we aim to further investigate activation and outcome of Ror2-dependent non-canonical Wnt signaling in breast cancer. To that end, we used the weakly invasive, estrogen receptor positive (ER+) breast cancer cell line MCF-7 as a model system for intervention experiments. The Ror2 receptor and the ligand Wnt5a were chosen as non-canonical Wnt pathway members for the perturbation of MCF-7 cells. To explore the effect of these perturbations, the invasive capacity of the cells was measured and the mRNA of the cell lines was profiled. The RNA sequencing (RNA-Seq) data were further analyzed in a bioinfomatic framework by integration with existing Wnt signaling networks. The resulting Ror2/Wnt module was further explored in independent gene expression data of breast cancer patients in order to verify the involvement of non-canonical Wnt signaling in metastasis development (for an overview of experimental procedure/workflow steps see Figure 1).

## Materials and Methods

### Public Data

#### Patient Gene Expression Data

The breast cancer patient data are a collection of 10 public microarray datasets measured on Affymetrix Human Genome HG-U133 Plus 2.0 and HG-U133A arrays. The datasets were retrieved from Gene Expression Omnibus (GEO) (14) data repository, accession numbers GSE25066, GSE20685, GSE19615, GSE17907, GSE16446, GSE17705, GSE2603, GSE11121, GSE7390, and GSE6532. Each dataset was processed using the RMA probe-summary algorithm (15), and only samples with metastasis (or distant relapse)-free survival annotation were selected. The datasets were combined together on the basis of HG-U133A array probe names and quantile normalization was used over all datasets.

Breast cancer molecular subtypes for the patient samples were predicted by fitting a single sample predictor as implemented in the *genefu* R-package (16, 17) at prediction strength threshold 0.5 using PAM50 intrinsic genes list (1).

#### Wnt Network Models

Four previously published network models (18) represent distinct Wnt signaling cascades: *canonical Wnt signaling, non-canonical Wnt signaling, inhibition of canonical Wnt signaling*, and *regulation of Wnt signaling* pathways. Briefly, these models were constructed based on data from multiple pathway databases as directed signaling graphs with nodes corresponding to genes and edges corresponding to activation or inhibition processes. The network models can be utilized also as simple gene sets consisting of respective graph node labels.

#### Kyoto Encyclopedia of Genes and Genomes (KEGG) Gene Sets

Kyoto Encyclopedia of Genes and Genome (19) pathway gene sets represented by gene symbols were downloaded in June 2014 from Molecular Signature Database (20) as part of the C2 curated gene sets collection version 4.

### Cell Lines, Transfections, and Stimulations

MCF-7 and MDA-MB231 breast cancer cells (DSMZ) were cultured in RPMI-1640 medium (PAA) supplemented with 10% heat-inactivated fetal calf serum (FCS; Sigma). Generation of MCF-7 cells overexpressing Ror2 is described in Ref. (21). Overexpression of hRor2 in MDA-MB231 cells was achieved by transfecting the cells with either the pcDNA3.1/Zeo(+) empty vector (Invitrogen) or an hRor2 overexpression vector (kindly provided by Alexandra Schambony) using the Nanofectin transfection reagent (PAA). Cells overexpressing hRor2 were selected with zeomycin (100 µg/ml). MDA-MB231 shRor1 cells were generated as described previously (21) and selected in culture medium with puromycin (2 µg/ml). Vectors for shRNA-mediated gene knockdown were purchased from Thermo Scientific. The mature targeting sequence for Ror1 was 5′-ATTTATAGGATCTGCCATG-3′. For stimulation experiments, cells were treated for 24 h with Wnt5a (100 ng/ml, R&D systems) prior to cell lysis.

### Western Blot and Flow Cytometry

The protocol for sub-cellular fractionation has been previously published (21). For analysis of protein expression, cells were lysed in RIPA buffer (150 mM NaCl/0.1% SDS/0.5% Na-deoxycholate/1% Triton X-100/50 mM Tris, pH 7.2) and up to 50 µg of protein were loaded onto SDS-PAGE gels (8–10%). Proteins were transferred to nitrocellulose membranes and detected with primary antibodies specific to P-JNK (#9251), HDAC1 (#2062), Ror1 (#4102), JNK (#9252, all from cell signaling), Ror2 (#sc-98486), total β-catenin (#sc-7963), RhoA (#sc-418), Pkc (#sc-10800, all from Santa Cruz), active β-catenin (#05-665), and Tubulin (#05-829, both from Merck Millipore), as well as matching secondary antibodies (Santa Cruz Biotechnology). Chemiluminescence was detected with ECL Prime (GE Healthcare) at the LAS-4000 Imager (Fujifilm). Signals were quantified by densitometry using ImageJ (version 1.41) and normalized on Tubulin expression.

Ror2 expression was measured with an Alexa Fluor 488-coupled anti-Ror2 antibody (#FAB20641G) and the respective isotype control (#IC003G, both R&D systems) at the FACS Canto II flow cytometer using the BD FACS Diva software (version 6.1.3., BD Biosciences). Overlays were created with GIMP (version 2.8).

### Immunohistochemistry

To investigate the hormone receptor status immunohistochemistry was performed. Therefore, cells were centrifugated and washed in PBS. Then, they were resuspended (1⋅10^−6^/ml PBS). 200 µl were placed on the object carrier and centrifuged at 800 RPM for 5 min and then dried. Estrogen (ER), progesteron (PR), and Her2 were determined from the routine histopathological workup using immunohistochemical staining. The monoclonal mouse anti-human ER α antibody (#1D5) as well as the monoclonal mouse anti-human PR antibody (#636, both DAKO, Denmark) were used at a dilution of 1:100 and the rabbit monoclonal Her2 antibody (#SP3, Thermo Scientific, UK) at a dilution of 1:200. For all three antibodies, a standardized immunohistochemical staining technique was performed including a 90-min heat epitope retrieval using the immunostainer followed by a 45-min incubation with the specific antibody.

### Microinvasion Assay

Cancer cell invasion was measured in a modified Boyden chamber (22). MCF-7 (1⋅10^5^) or MDA-MB231 (5⋅10^4^) cells were seeded in triplicates onto an ECM-coated (R&D systems) polycarbonate membrane (pore diameter: 10 µm, Nucleopore), stimulated with or without Wnt5a (100 ng/ml, R&D systems) and incubated for 96 h at 37°C. The number of invasive cells in the lower wells was counted and related to the unstimulated control.

### RNA Deep Sequencing

Library preparation for RNA-Seq was performed using the TruSeq Stranded Total RNA Sample Preparation Kit (Illumina, RS-122-2201) starting from 1,000 ng of total RNA. Accurate quantitation of cDNA libraries was performed by using the QuantiFluor TM dsDNA System (Promega). The size range of final cDNA libraries was determined applying the SS-NGS-Fragment 1–6,000 bp Kit on the Fragment Analyzer from Advanced Analytical (320 bp). cDNA libraries were amplified and sequenced by using the cBot and the HiSeq2000 from Illumina (SR; 50 bp; 35 million reads per sample). Sequence images were transformed with Illumina software BaseCaller to bcl files, which were demultiplexed to fastq files with CASAVA v1.8.2.

### Quantitative Real-time PCR (qRT-PCR)

Total RNA from empty vector (pcDNA) and Ror2-overexpressing (pRor2) cells was extracted using the High Pure RNA isolation kit (Roche). For each sample, 1 µg of RNA was transcribed into cDNA with the iscript cDNA synthesis kit (Bio-Rad). Gene expression was measured by SYBR green detection on the ABI PRISM 7900HT system (Applied Biosystems) from 10 ng cDNA per reaction with gene-specific primers. Data were analyzed with the SDS software version 2.4. (Applied Biosystems) and target gene expression quantified with the ΔΔct-method after normalization to the two housekeeping genes *HPRT1* and *GNB2L1*. Primer sequences are as follows:

Ror2: fw_5′-TTCTTCTTGGTTTGCATGTG-3′, rv_5′-CTGATCTCTTTGAGTTTGGC-3′
HPRT1: fw_5′-TATGCTGAGGATTTGGAAAGG-3′, rv_5′-CATCTCCTTCATCACATCTCG-3′
GNB2L1: fw_5′-AACCCTATCATCGTCTCCT-3′, rv_5′-CAATGTGGTTGGTCTTCAG-3′
LCP-1: fw_5′-GCGGACATTTAGGAACTGGA-3′, rv_5′-GTATGGCGGTTTGTTTACTCTG-3′
FAT-1: fw_5′-TCCTCCTGACATCATCTGCC-3′, rv_5′-GATAGATGCTCTCCTCAATTACCC-3′
VIL-1: fw_5′-ATGAGCAGGAGAAGAAGGGA-3′, rv_5′-TCATTCTGCACCTCCACCT-3′
WIPF1: fw_5′-GAAATGGCTTCCAAGACTCTC-3′, rv_5′-GTAGAATCTGCTTTCCCACTC-3′
HNF4G: fw_5′-TATAGACTCCGTTCCCTACCA-3′, rv_5′-TTTCCTGTTGCTCTGTCCC-3′

### Statistical and Bioinformatic Analyses

#### RNA-Seq Processing and Differential Analysis

RNA sequencing data were first quality checked *via* FastQC (Babraham Bioinformatics). The reads were then mapped against the reference genome GRCh37 with the STAR RNA-Seq alignment tool (23), while incorporating database information from Ensembl ver. 37.73 during the reference indexing step. Gene-level abundances were estimated using the RSEM algorithm (24). Further processing steps were performed using the *edgeR* (25) R-package. Non-expressed genes were filtered out by keeping the genes with at least one count-per-million reads in at least three samples. Differential genes between different conditions were identified by fitting negative binomial generalized linear models (26). Gene *p*-values were adjusted for multiple testing using Benjamini–Hochberg method (27) resulting in false-discovery rate (FDR) values and significantly differentially expressed genes (DEGs) were considered at FDR < 0.05 level. The raw RNA-Seq data have been submitted to GEO repository under the accession number GSE74383 for MCF-7 conditions and under the accession number GSE96637 for MDA-MB231 conditions.

#### Gene Set Enrichment and Network Integration Analyses

Differential targets identified in the analysis of RNA-Seq data were further subjected to enrichment and network integration analyses. To test enrichment of pathways, a simple gene set approach was applied (28). In particular, over-representation of the common target genes was tested using Fisher’s exact test (29), whereas rank-based enrichment testing of a full list of gene *p*-values was performed using Wilcoxon rank-sum test.

The network integration analysis steps were performed as described in Ref. (30). In brief, the common targets were first mapped onto the nodes of the *non-canonical Wnt signaling* network model, and the nodes induced by the mapped targets were used as terminal nodes for the Steiner tree analysis as implemented in the *SteinerNet* R-package (31). In this analysis the Steiner tree, minimal size subgraph connecting all terminal nodes, is searched within the undirected network based on shortest path approximation and so-called Steiner nodes are introduced to ensure connectivity. All nodes of the Steiner tree were used to extract an induced subnetwork containing all original directed edges. For visualization purpose the range for the node color coding was limited to ±2-fold change.

#### Clustering and Survival Analyses

For the analysis of public gene expression data of breast cancer patients, the complete-linkage hierarchical clustering was performed based on Pearson correlation as the distance measure. When multiple probes corresponded to a single gene, the probe with highest average expression level was used to represent a gene in the clustering analysis.

The patient samples were clustered based on a gene signature originating from the network integration analysis. Distinct patient clusters within the dendrogram were identified using dynamic hybrid cut algorithm implemented in the function *cutreeDynamic* from the *dynamicTreeCut* R-package (32). The clusters were detected in a bottom-up manner based on the dendrogram shape and the correlation dissimilarity information among the patients. The minimum cluster size parameter was set as 12.5% of the patients when the whole dataset was clustered and 25% of the patients when only patients of a particular molecular breast cancer subtype were clustered.

Resulting patient clusters were subjected to a Kaplan–Meier (KM) analysis of metastasis-free survival (MFS). KM curves were compared using a log-rank test implemented the in *survival* R-package (33). When plotting the KM curves the first 15 years were visualized.

#### Clustering and Survival Analyses Based on Random Signatures

For control purposes, significance of 1,000 random signatures was investigated in the same manner as the original signature from network integration analysis. Random signatures were generated by sampling 76 genes from the pool of 4,140 KEGG pathway’s genes for 1,000 times. The gene pool was created by merging all KEGG pathway gene sets and by limiting the pool to the unique genes which are represented by a HG-U133A array probe in the patient gene expression dataset. Subsequently, all steps of hierarchical clustering, detection of clusters in the dendrogram using dynamic hybrid cut algorithm, and MFS analysis were repeated for each of 1,000 random signatures.

## Results

### Ror2 Overexpression Enhances Invasiveness of MCF-7 Cells

**Figure 2:**
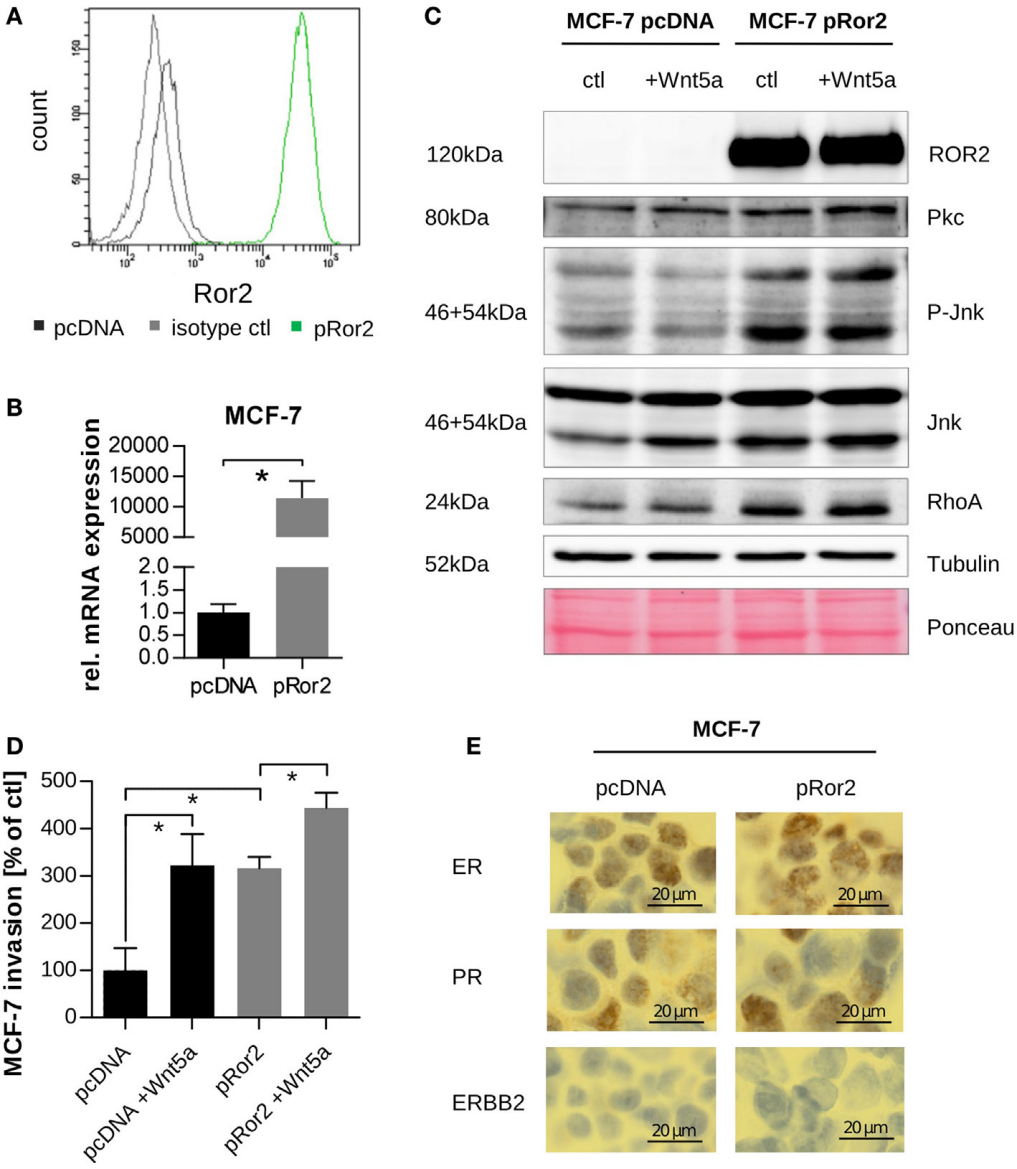
Overexpression of Ror2 in MCF-7 cells activates non-canonical Wnt signaling and enhances tumor invasion. **(A)** Flow cytometry: Ror2 expression was analyzed in MCF-7 cells transfected with an empty vector (pcDNA, black) or a Ror2 overexpression construct (pRor2, green). The isotype control (ctl) is shown in gray. **(B)** Quantification of *ROR2* gene expression by quantitative real-time PCR (*n* = 3, mean ± SD, *p < 0.0001). **(C)** Expression of non-canonical Wnt target proteins in pcDNA and pRor2 cells ± Wnt5a (100 ng/ml, 24 h) was assessed by western blotting. **(D)** Invasiveness of MCF-7 pcDNA and pRor2 cells with or without Wnt5a stimulation (100 ng/ml) was measured in a modified Boyden chamber (mean ± SD, *n* = 3, *p < 0.001). **(E)** Receptor status of MCF-7 pcDNA and pRor2 cells was analyzed by immunohistochemistry. One representative image is shown for each staining and condition.

To investigate the effect of non-canonical Wnt signaling on cancer progression and downstream signaling, the Wnt5a ligand and the membrane receptor Ror2 were chosen as non-canonical Wnt pathway members to be perturbed. In particular, MCF-7 cells were stably transfected either with an empty vector (pcDNA) or with a Ror2 overexpression construct (pRor2) and successful transfection was confirmed by flow cytometry (Figure 2A), qRT-PCR (Figure 2B), and western blotting (Figure 2C). Overexpression of Ror2 led to an activation of non-canonical Wnt signaling in MCF-7 cells with an increase in PKC and RhoA expression as well as Jnk phosphorylation (Figure 2C; Figure S1 in Supplementary Material). Wnt5a stimulation increased total JNK levels in control cells; however, it had no additional stimulatory effect in Ror2-overexpressing cells.

Moreover, elevated expression of Ror2 also increased MCF-7 invasiveness (Figure 2D). The same effect was observed when empty vector cells were stimulated with Wnt5a. Interestingly, the combination of both, Ror2 overexpression and additional stimulation with its ligand Wnt5a (pRor2 + Wnt5a condition), was able to even further enhance cancer cell invasion compared to the Ror2 overexpression alone (Figure 2D). This suggests that at least a part of the pro-invasive effect of Wnt5a is mediated through the Ror2 receptor.

Since it has been reported that under distinct stimulation MCF-7 cells can change their phenotype from hormone receptor positive to triple negative (34), we investigated whether this is true for Ror2 overexpression and might explain the gain in cell invasiveness. However, we did not detect any changes in the hormone status of the cells as analyzed by immunohistochemistry (Figure 2E). Therefore, we decided to characterize in depth the gene expression profiles of the cells with induced Ror2 overexpression and Wnt5a stimulation in order to identify novel targets, which might be involved in the increased invasiveness of the cells. The following four conditions were selected for further analysis by RNA-Seq: control MCF-7 cells with the empty vector (pcDNA), cells stimulated with Wnt5a (pcDNA + Wnt5a), cells with stable overexpression of Ror2 (pRor2), and a combination of both perturbations (pRor2 + Wnt5a).

### mRNA Profiling Reveals Targets of Ror2 Overexpression

To quantify the gene expression changes linked with the observed pro-invasive effects of Wnt5a and Ror2 in MCF-7 cells, each of the four conditions was profiled in three replicates using RNA-Seq. The library size of the sequenced samples ranged from 35 to 55 million (Table 1). In the differential analysis, gene expression profiles of the different conditions were compared to identify downstream targets of the distinct perturbations. Therefore, five comparisons (Table 2) were performed to identify DEGs.

**Table 1:** Deep sequenced MCF-7 samples and size of libraries.

Condition of MCF-7 cells	Rep.	Library in millions
pcDNA	1	50
pcDNA	2	53
pcDNA	3	47
pcDNA + Wnt5a	1	35
pcDNA + Wnt5a	2	55
pcDNA + Wnt5a	3	49
pRor2	1	48
pRor2	2	36
pRor2	3	53
pRor2 + Wnt5a	1	49
pRor2 + Wnt5a	2	51
pRor2 + Wnt5a	3	35

*The table summarizes RNA-Seq sample replicates (Rep.) of the perturbation experiments on MCF-7 cells with the total number of the mapped reads in million*.

**Table 2:** Comparisons in the differential analysis.

Tested effect	Comparison	Number of DEGs	List of DEGs
Wnt5a	pcDNA vs. pcDNA + Wnt5a	7	Table S1 in Supplementary Material (sheet 1)
Wnt5a	pRor2 vs. pRor2 + Wnt5a	11	Table S1 in Supplementary Material (sheet 2)
Ror2	pcDNA vs. pRor2	2,860	Table S2 in Supplementary Material (sheet 1)
Ror2 + Wnt5a	pcDNA vs. pRor2 + Wnt5a	3,729	Table S2 in Supplementary Material (sheet 2)
Ror2	pcDNA + Wnt5a vs. pRor2 + Wnt5a	3,022	Table S2 in Supplementary Material (sheet 3)

*Differentially expressed genes (DEGs) were identified by comparing expression profiles of MCF-7 cells with different perturbations*.

**Figure 3:**
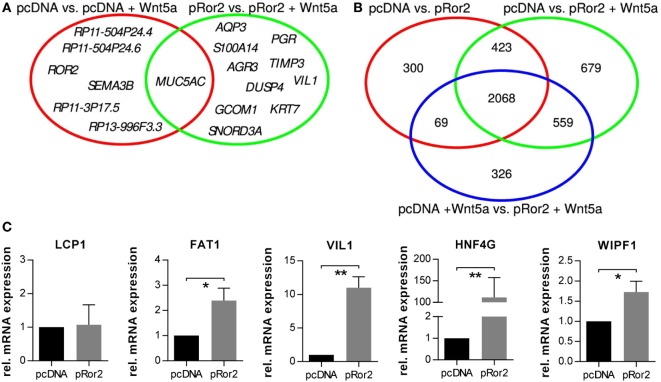
Venn diagrams of perturbation targets. **(A)** Wnt5a targets: overlap of significantly differentially expressed targets of Wnt5a stimulation in the MCF-7 cells with and without Ror2 receptor overexpression. **(B)** Ror2 targets: overlap of three lists of significant differentially expressed genes responsive to the Ror2 overexpression in MCF-7. **(C)** Gene expression changes of the top five genes regulated in Ror2-overexpressing vs. empty vector MCF-7 cells were validated by quantitative real-time PCR (*n* = 3, mean ± SD, *p < 0.05, **p < 0.01).

The two comparisons testing for the effect of the Wnt5a stimulation, with and without presence of the overexpressed Ror2 (pcDNA vs. pcDNA + Wnt5a and pRor2 vs. pRor2 + Wnt5a), yielded rather low numbers of significantly DEGs (Figure 3A; Table S1 in Supplementary Material). The single significant DEG, which was detected in the both comparison, was *MUC5AC*. These low numbers of DEGs indicated that Wnt5a stimulation had only moderate effect on the gene expression changes of the MCF-7 cell line and suggest that it mediates its pro-invasive effects rather on the protein level.

The three comparisons that tested for the impact of the Ror2 overexpression (pcDNA vs. pRor2, pcDNA vs. pRor2 + Wnt5a, pcDNA + Wnt5a vs. pRor2 + Wnt5a) demonstrated the strongest effects resulting in 2,860, 3,729, and 3,022 DEGs, respectively (Table S2 in Supplementary Material). Stable targets of Ror2 overexpression were determined in a Venn analysis as an overlap of these three differential gene lists that resulted in 2,068 common targets (Figure 3B). We selected the top five genes from this overlap to validate the observed gene expression changes by qRT-PCR. Indeed, we were able to detect a significant upregulation of *FAT1, VIL1, HNF4G*, and *WIPF1* in Ror2-overexpressing MCF-7 cells compared to control cells, whereas the upregulation of *LCP1* was not confirmed (Figure 3C).

### Ror2 Targets Are Enriched in Non-Canonical but Not in Canonical Wnt Gene Set

To explore the gene list of 2,068 Ror2 targets in the context of different Wnt signaling cascades, we performed enrichment analysis. Four Wnt models representing distinct Wnt signaling pathways were used as gene sets for enrichment testing. To further scrutinize the contribution of the upregulated and downregulated genes to the enrichment, the targets were sorted based on positive and negative fold-changes into three lists: *all, up*, and *down*. Two Wnt pathways were detected as over-represented in the list of all targets: non*-canonical Wnt signaling* and *Regulation of Wnt signaling* (Table 3). Whereas the *Non-canonical Wnt signaling* gene set was significant for *all* target list as well as for upregulated targets, the *Canonical Wnt signaling* gene set was not significant for any target list.

**Table 3:** Over-representation of Ror2 overexpression targets in the four Wnt gene sets.

WNT gene set	2,068 common targets		
	All	Up	Down
Can.	0.35	0.30	0.54
Non-can.	**0.02**	**0.002**	0.65
Inh.	0.98	0.91	0.97
Reg.	**0.01**	0.09	**0.04**

*Fisher’s exact test was performed to test for over-representation of 2,068 targets of Ror2 overexpression in the four distinct Wnt gene sets*.

*Significant *p*-values (*p* < 0.05) are depicted in bold*.

### Ror2 Targets Affect Cell Remodeling Processes and Cell Metabolism

**Figure 4:**
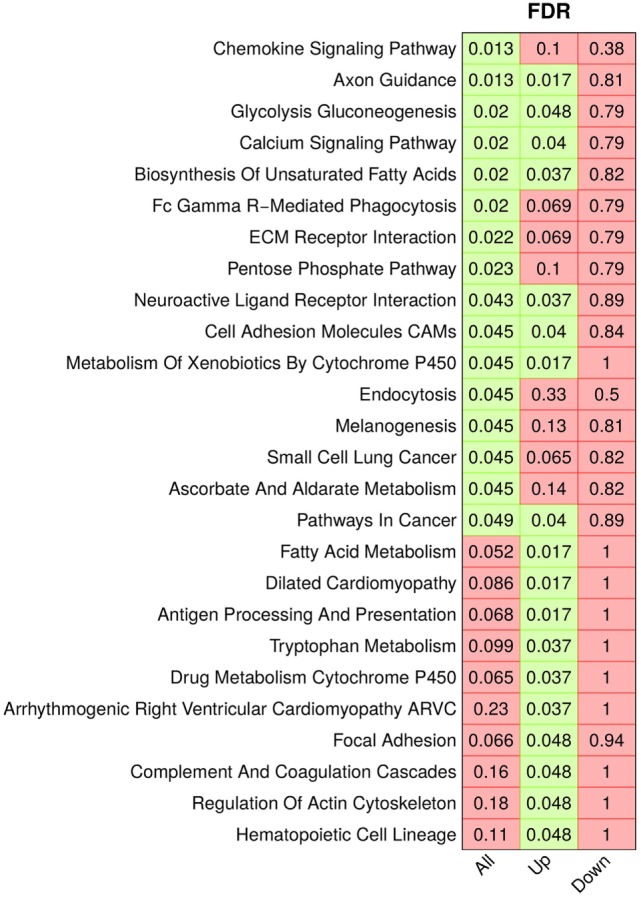
Enrichment analysis of KEGG pathways. Adjusted *p*-values (*q*-values) depicting significant enrichment (*q*-value < 0.05, green) of the gene sets in the three target lists: all targets (All), upregulated targets (Up), and downregulated targets (Down). Non-significant values are displayed in red.

We further investigated the target list in an enrichment analysis beyond the context of Wnt signaling, exploring other signaling and metabolic processes which were altered by Ror2 overexpression. We tested pathway gene sets from the KEGG database and identified 16 pathways enriched in the *all* list, 18 pathways enriched in the *up* list and no pathway enriched in the *down* list (Figure 4). This resulted in a collection of 26 pathways affected by Ror2 overexpression, of which eight pathways were enriched in both the *up* and the *all* list. This collection comprised multiple pathways modulating cell metabolism (e.g., *glycolysis gluconeogenesis, pentose phosphate pathway, fatty acid metabolism*) as well as pathways directly or indirectly involved in cell remodeling and migration processes (e.g., *ECM receptor interaction, regulation of actin cytoskeleton, chemokine signaling pathway*).

### Network Integration Reveals a Ror2/Wnt Module: Ror2-Expression-Responsive Subnetwork of Non-Canonical Wnt Pathway

The list of 2,068 targets was further used for network integration analysis. The results of the Wnt pathways enrichment analysis suggested activation of non-canonical Wnt signaling in the gene expression data. Therefore, the non-canonical Wnt network model was chosen for the subsequent network integration in order to identify a module induced by the Ror2 overexpression targets. The underlying non-canonical Wnt model is a signaling network of 489 nodes representing pathway genes interconnected by activation and inhibition edges.

**Figure 5:**
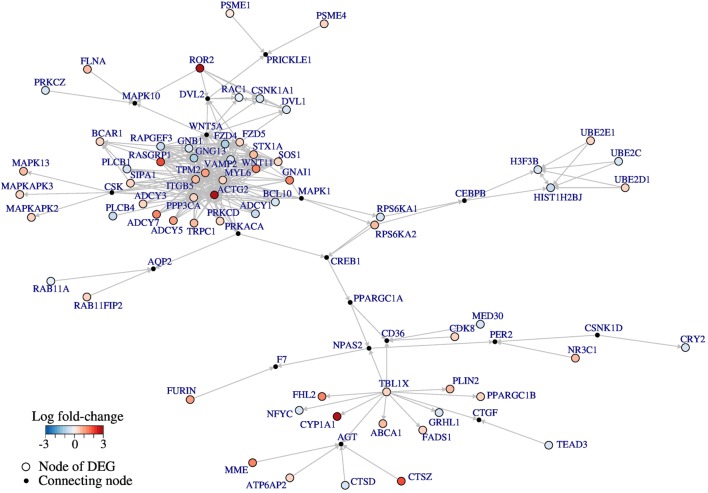
Ror2-expression-responsive module. Non-canonical Wnt subnetwork representing a differentially regulated module of the Wnt pathway. The color coding corresponds to the fold-changes of the targets: blue represents downregulation and red upregulation of the genes in Ror2-overexpressing cells compared to the control MCF-7 cells. The smaller black nodes were introduced by Steiner tree analysis. The directed edges represent controlling interactions.

First, 2,068 target genes were mapped onto the nodes of the non-canonical Wnt network, which resulted in 66 induced nodes. To link these induced nodes within the network structure, the Steiner tree algorithm was employed. In this step, 18 connecting nodes, so-called Steiner nodes, were introduced that do not embody differential targets. Subsequently, the induced subnetwork including all original edges between the 84 nodes was extracted (Figure 5; Table S3 in Supplementary Material). This subnetwork represents the module of non-canonical Wnt pathway regulated by the overexpressed Ror2 receptor (hereinafter referred to as *Ror2/Wnt module*). The Ror2/Wnt module revealed several important Wnt pathway members: differentially regulated ligand *WNT11*, receptors *FZD5* and *FZD4*, and signal transducer *DVL1*; as well as *WNT5A, DVL2*, and *CD36* as Steiner nodes interconnecting the differential targets.

### Predicted Breast Cancer Molecular Subtypes Show Metastatic Differences

We considered the members of the Ror2/Wnt module to be candidate genes of the non-canonical Wnt pathway that confer an aggressive phenotype to MCF-7 breast cancer cells after Ror2 overexpression. Therefore, we further aimed to assess the impact of the Ror2/Wnt module genes in the clinical context of metastatic breast cancer. To this end, we first collected available breast cancer patient data of expression profiles annotated with MFS follow-ups.

Ten public gene expression datasets of patient samples were assembled into a compendium dataset. Annotations of a metastasis event with time to metastasis (or distant recurrence/distant relapse) information were compiled together for 2,075 patients. In this cohort, the molecular breast cancer subtypes were predicted using the PAM50 gene signature. For 1,724 patients (out of 2,075) one of the following subtypes was assigned: Basal, LumA, LumB, or Her2 (Table 4). As no sample was predicted as normal-breast-like subtype above the prediction strength threshold, we did not consider this subtype for further analyses.

**Table 4:** Summary of metastasis events by predicted subtypes.

Subtype	No of patients	No of events (%)	5 years MFS
Basal	289	74 (0.26)	0.74
ERBB2-overexpressing	47	18 (0.38)	0.61
LumA	716	116 (0.16)	0.92
Luminal B	672	186 (0.28)	0.76
All subtypes	1,724	394 (0.23)	0.82

*Table summarizes for the whole dataset as well as for each subtype separately the number (No) of patients, the number of metastasis events, the percentage of the metastasis events within a group, and the 5-year metastasis-free survival (MFS) rate*.

As the molecular breast cancer subtypes are known to have different prognoses, we investigated differences in MFS among predicted subtypes as a quality benchmark step. We identified the highest 5-year MFS rate of 0.92 for the LumA patients, whereas for the Basal-like and Her2 subtype patients the rate was the lowest—0.74 and 0.61, respectively (Table 4). We further tested the KM curves of predicted patient groups and showed prognostic significance of breast cancer subtypes in terms of developing metastasis (Figure S2 in Supplementary Material).

### Translation of Ror2/Wnt Module Genes to Breast Cancer Patient Data

**Figure 6:**
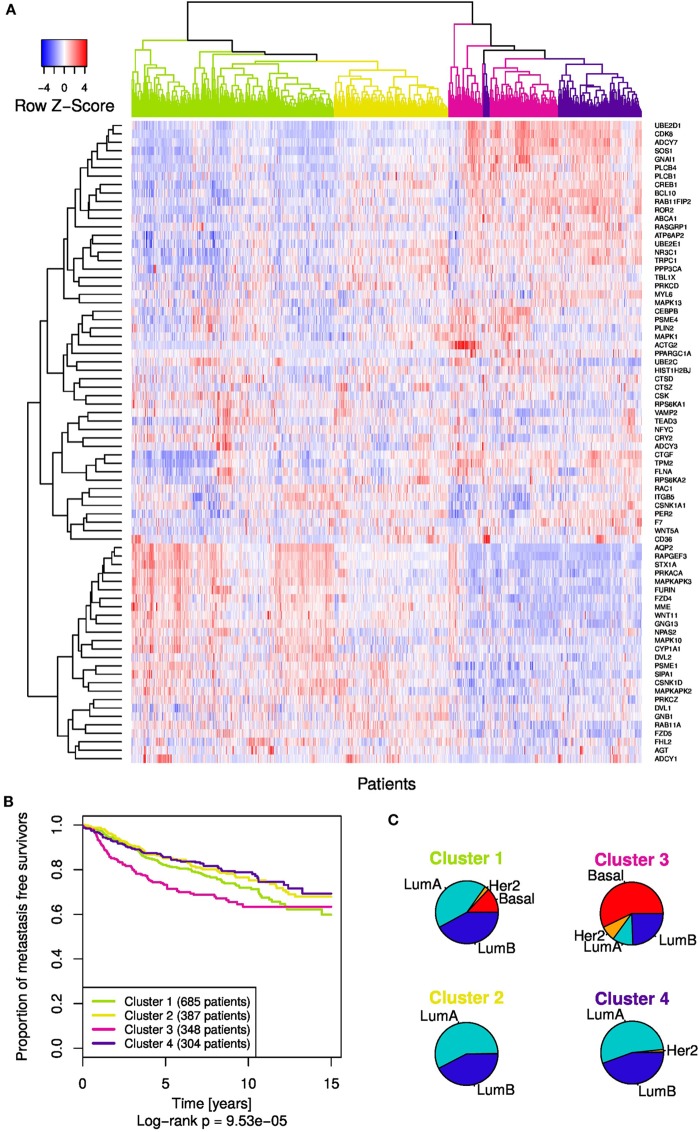
The Ror2/Wnt module gene signature in the whole patient cohort. **(A)** Clustering analysis revealed heatmap of expression levels (depicted as row *z*-scores) and the subsequent dendrogram shape-based analysis yielded four patient clusters. **(B)** Kaplan–Meier curves showing metastasis-free survival according to the four patient clusters. **(C)** The pie charts display distribution of the subtypes within each cluster.

The identified 84 genes in the Ror2/Wnt module were used as a pathway-based gene signature to assess prognostic power for metastasis development in breast cancer. Out of these 84 genes, 76 could be mapped to the patient expression data. Expression levels of these 76 genes of the Ror2/Wnt module were utilized for the correlation distance-based clustering analysis (Figure 6A). To determine the number of clusters in the patient dendrogram, the dynamic hybrid algorithm was employed and identified four distinct clusters. These four clusters exhibited significant differences in MFS (Figure 6B) with the *Cluster 3* (magenta) having markedly worse prognosis. This cluster comprised a majority of Basal subtype patients; however, all clusters contained mixtures of at least two or more subtypes (Figure 6C).

**Figure 7:**
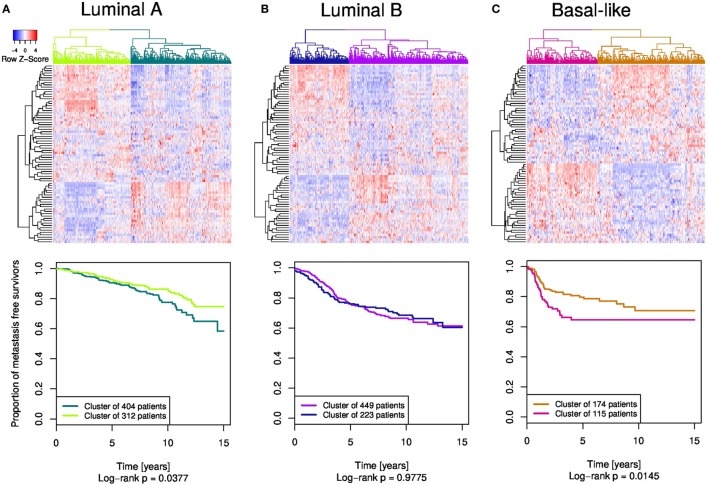
The Ror2/Wnt module gene signature applied in **(A)** luminal A, **(B)** luminal B, and **(C)** basal-like subtypes. Subtype-specific heatmaps are displayed at the top, whereas associated Kaplan–Meier curves depicting metastasis-free survival are at the bottom.

The mixed distribution of breast cancer subtypes across the four patient clusters in the dendrogram motivated us to explore the MFS within the individual subtypes in regard to the Ror2/Wnt module expression patterns. Therefore, we performed subtype-specific patient clustering followed by KM analysis of MFS, based on the same Ror2/Wnt module-based gene signature as previously done for the whole cohort. Clustering and cluster-detection analyses revealed two clusters in each of the patient groups of LumA, LumB, and Basal subtypes (Figure 7; Figures S3–5 in Supplementary Material). The Her2 subtype was not included due to the relatively small number of patients.

For the LumA and Basal subtype the patient subgroups showed significant differences in MFS (*p* = 0.0377 and *p* = 0.0145, respectively). In contrast, two subgroups detected within the LumB subtype showed no difference in MFS (*p* = 0.9775). LumA subtype patients grouped in the cluster of 312 samples (light-green) had better prognosis than the 404 patients in the second LumA cluster (deep-sea-blue). In the Basal subtype-specific analysis the two clusters exhibited a significant difference with the KM curve of the smaller patient cluster (115 samples, magenta) showing worse metastasis prognosis than the bigger cluster of 174 patients (brown).

**Figure 8:**
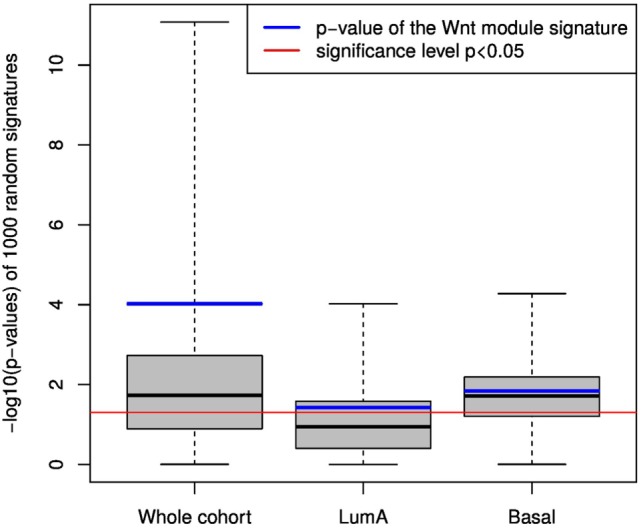
Performance of random signatures compared to Ror2/Wnt module gene signature. The boxplots visualize log-transformed *p*-values from metastasis-free survival analysis of 1,000 random signatures in the whole cohort and lumA and Basal subtypes. Blue bars display *p*-values of the original Ror2/Wnt module-based gene signature. Red line depicts significance level *p* = 0.05.

Furthermore, we compared the performance of Ror2/Wnt module genes signature to the prognostic performance of random signatures in the same data. This step was taken in order to investigate prognostic superiority of the original signature over random ones and thus to ascertain its clinical relevance. Thousand gene signatures of the same size as the original (76 genes) were randomly sampled from the pool of 4,140 genes from KEGG pathways. Thereon, the analysis pipeline of hierarchical clustering, automatic detection of patient clusters, and KM analysis of MFS was executed. For each random signature the pipeline yielded a log-rank *p*-value that describes the significance of difference in MFS in detected patient groups. The same set of 1,000 random signatures was applied to the whole cohort as well as to LumA and Basal subsets. The resulting *p*-values were log-transformed (−*log10*) and visualized together with the corresponding *p*-values of the original Ror2/Wnt module gene signature (Figure 8).

From the 1,000 random signatures 63.2, 34.9, and 69.9% were detected as significantly prognostic (*p* < 0.05) in the whole cohort, the LumA group and the Basal group, respectively. Further, we checked the percentage of random signatures that performed better than the original Ror2/Wnt module-based signature: in the whole patient cohort 9% of the random signatures were more strongly associated with MFS than the original signature (*p* = 9.53e−05). In the groups of LumA and Basal subtypes, 30.5 and 42.9% of random signatures outperformed the original (*p* = 0.0377 and *p* = 0.0145), respectively.

### Knockdown of Ror1 in MDA-MB231 Cells Decreases Non-Canonical Wnt Signaling

**Figure 9:**
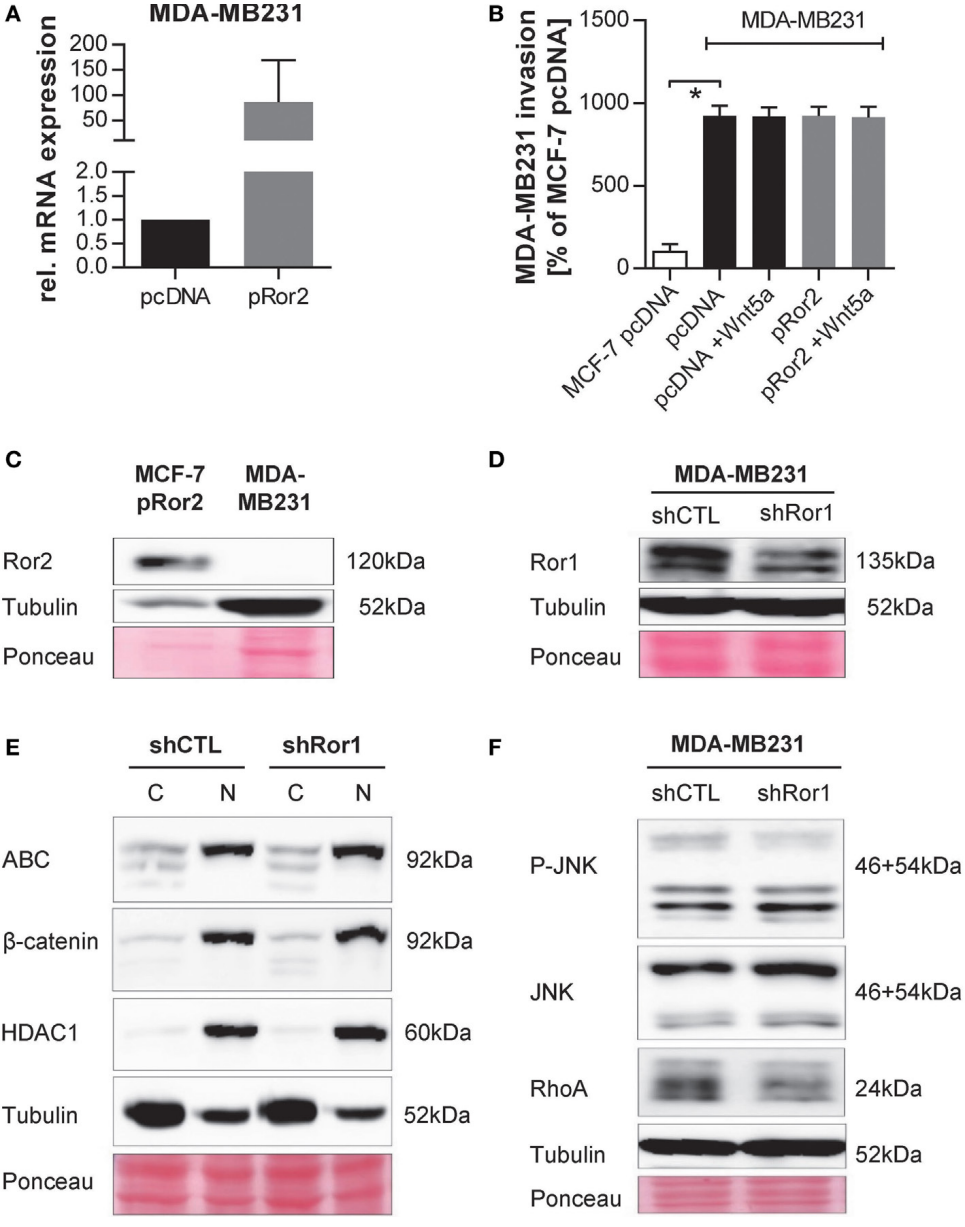
Knockdown of Ror1 in MDA-MB231 cells decreases non-canonical Wnt signaling. **(A)** Ror2 expression was measured in MDA-MB231 cells expressing an empty vector (pcDNA) or hRor2 overexpression construct (pRor2) by quantitative real-time PCR (*n* = 3, mean ± SD). **(B)** Cell invasion assay of MDA-MB231 pcDNA and pRor2 cells ± Wnt5a (100 ng/ml). Invasion rates are shown in relation to invasion of MCF-7 pcDNA cells (mean ± SD, *n* = 4, *p < 0.0001). **(C)** Western blot of endogenous Ror2 expression in MDA-MB231 cells. MCF-7 pRor2 cells are shown as positive control. **(D)** Western blot of Ror1 expression in MDA-MB231 non-sense control (shCTL) and shRor1 cells. Ponceau staining and Tubulin are depicted as loading controls. **(E)** MDA-MB231 shCTL and shRor1 cells were fractionated and expression of active β-catenin (=ABC) as well as total β-catenin analyzed in cytoplasm (=C) and nucleus (=N) by Western Blotting. HDAC1 expression was analyzed to confirm successful fractionation. **(F)** Western Blot: MDA-MB231 shCTL and shRor1 cells were characterized for non-canonical Wnt target proteins.

In order to investigate whether Ror2 and Wnt5a also have pro-invasive effects in triple-negative MDA-MB231 cells, we overexpressed Ror2 in the cells and confirmed successful transfection by qRT-PCR (Figure 9A). However, cell invasion assays showed that, in contrast to MCF-7, MDA-MB231 cells are already highly invasive and cannot be stimulated any further neither by Ror2 overexpression nor by addition of Wnt5a (Figure 9B). Interestingly, MDA-MB231 cells do not express any endogenous Ror2 (Figure 9C); however, it has been shown previously that instead they express its family member Ror1, which is important for the invasive phenotype of the cells (21). Therefore, we performed a stable knockdown of Ror1 in these cells (Figure 9D). While this had no effect on canonical Wnt signaling (Figure 9E), RhoA levels and JNK phosphorylation were decreased in the knockdown cells (Figure 9F).

Similar to the MCF-7 cells, we were interested in a large-scale identification of the downstream gene expression changes and, therefore, these two conditions were selected for RNA-Seq: MDA-MB231 cells transfected with a non-silencing shRNA (shCTL) and cells transfected with a Ror1 shRNA (shRor1). The library size of the sequenced samples ranged from 47 to 60 million (Table 5). However, in the differential gene expression analysis comparing shCTL vs. shRor1 samples we identified only two significant DEGs: proto-oncogene AGR2 and a gene for uncharacterized protein RP11-1012A1.4. Despite of this low number of DEGs, we aimed to further explore the entire lists of measured genes in the context of distinct Wnt signaling cascades using rank-based gene set enrichment procedures. We detected significant enrichment of the *Non-canonical Wnt signaling* gene set (*p* = 0.035) and the *Inhibition of canonical Wnt signaling* gene set (*p* = 0.002), whereas *Canonical Wnt signaling* (*p* = 0.056) and *Regulation of Wnt signaling* (*p* = 0.870) gene sets were not significant. This indicates that although the expression changes after Ror1 knockdown were only moderate, the decreased invasiveness could be associated with altered activity of the non-canonical Wnt signaling.

**Table 5:** Deep sequenced MDA-MB231 samples and size of libraries.

Condition of MDA-MB231 cells	Rep.	Library in millions
shCLT	1	60
shCLT	2	55
shCLT	3	47
shRor1	1	56
shRor1	2	40
shRor1	3	49

*The table summarizes RNA-seq sample replicates (Rep.) of the perturbation experiment on MDA-MB231 cells with the total number of the mapped reads in million*.

## Discussion

Activation states of canonical and non-canonical Wnt signaling pathways in breast cancer have so far eluded detection. Based on the previous results of Klemm et al. (11), we hypothesized that the non-canonical Wnt pathway is critical for progression of breast cancer. Here, we performed pathway interventions at the ligand and membrane receptor level in order to elucidate mechanism and outcome of this signaling cascade. In particular, we used the weakly invasive ER-positive MCF-7 cells which were transfected with an empty or Ror2 overexpression vector and optionally stimulated with recombinant Wnt5a in parallel. At the phenotypic level, the major consequence of the individual as well as combined perturbations was increased cell invasion. Therefore, to explore the large-scale effects of these perturbations at the gene expression level, the mRNA of the cells was sequenced and DEGs were identified.

Studies on the role of Wnt5a in breast cancer reported contradicting evidence, with Wnt5a either enhancing or suppressing invasiveness of different breast cancer cells (35). Here we showed that Wnt5a has a clear pro-invasive effect on the MCF-7 cells. However, the numbers of differential targets of Wnt5a stimulation identified in RNA-Seq data were rather low (up to 11 genes). As we could not observe major changes in gene expression, the Wnt5a ligand could potentially mediate the signals leading to the phenotypic changes by activation of proteins in the PCP pathway (36) as opposed to the transcription of new genes. The top ranked differentially expressed target, which was upregulated after Wnt5a stimulation, is the *ROR2* gene. Wnt5a is known to bind Ror2 (37) and this Ror2 upregulation demonstrates a possible positive feedback loop. The single differential gene common for both comparisons testing for Wnt5a stimulation effect in MCF-7 was *MUC5AC*, mucin 5AC. This gene has been studied in the context of colorectal (38) as well as pancreatic cancer (39), and in the latter cancer type its expression was associated with tumor growth. However, to our best knowledge, expression of *MUC5AC* has so far neither been linked to invasive breast cancer nor been reported as a potential target of Wnt5a signaling.

The Ror2-overexpressing cells also showed a significant increase in their invasiveness compared to the control cell line. Similar observations have not only been made for MCF-7, but also for Her2-positive SK-BR-3 cells (21), thus pointing to a general effect of Ror2 on breast cancer cell invasiveness. However, invasion of triple-negative MDA-MB231 cells was not enhanced further by Ror2 overexpression, probably due to their already high invasive potential. In contrast, knockdown of Ror1, which is highly expressed endogenously in these cells, diminished non-canonical Wnt signaling as shown by Western Blotting and reduces the invasive potential of the cells (21). Although by RNA-Seq we have not detect any major gene expression changes between MDA-MB231 control cells and Ror1 knockdown, we identified enrichment of non-canonical Wnt signaling gene set which could be associated with previously observed decrease of MDA-MB231 cells invasiveness. Interestingly, both Ror1 and Ror2 have been suggested as receptors for Wnt5a (40), have been linked to breast cancer progression and their expression was previously observed in breast cancer brain metastasis (11).

Combined treatment of MCF-7 cells with both Wnt5a stimulation and Ror2 overexpression exhibited even a stronger pro-invasive effect than the single stimulations. We assume that by presence of both the ligand as well as its receptor the non-canonical Wnt5a/Ror2 signaling cascade was highly stimulated (37), which then further drove the invasiveness. However, without Wnt5a stimulation the MCF-7 cells express no or low levels of Wnt5a (11) and there were no changes detected in *WNT5A* gene expression levels after Ror2 overexpression. This opens an intriguing question about which other ligand could have mediated the signaling *via* Ror2 and the subsequent cell invasion in the Ror2-overexpressing cells. An interesting candidate could be Wnt11. Although it is unclear whether the Wnt11 protein was present at the time of perturbation, the *WNT11* gene was subsequently transcribed and could potentially act as a ligand mediating non-canonical signals *via* Ror2. However, Wnt11/Ror2 signaling has been described only in zebrafish (41) and is not known in humans so far. Nevertheless, Wnt11 itself has been reported to be involved in tumor progression of several cancer types (42–45).

At the transcriptomic level, the three differential comparisons that tested for the Ror2 overexpression targets demonstrated, in contrast to the low number of genes affected by the Wnt5a exposure, a stronger effect of this perturbation. The overlap of the three gene lists revealed 2,068 common DEGs, which represent stable targets of Ror2 overexpression independent on whether the Wnt5a stimulation was present or not. We consider these common targets to be candidate genes that confer the invasive phenotype to MCF-7 breast cancer cells. To gain further insight into the biology underlying this fairly long list of expression-responsive Ror2 targets, we performed enrichment and network integration analyses.

For testing the enrichment of Wnt signaling gene sets and KEGG pathways in the common targets list the over-representation analysis approach was utilized. In the context of four different Wnt gene sets, the one representing the *Non-canonical Wnt signaling* pathway was detected as significant in the all target list as well as in the sublist of only upregulated targets. This suggests that the Ror2 overexpression induces activation of non-canonical Wnt signaling, which is in accordance with the upregulation and/or activation of several non-canonical Wnt proteins that we observed in the MCF-7 Ror2-overexpressing cells by Western Blotting. Beside the non-canonical Wnt gene set, the *Regulation of Wnt signaling* gene set was significantly over-represented in the target list, which indicates that Ror2 overexpression also modulates activity of the pathways acting upstream of the Wnt ligands.

Enrichment analysis of KEGG gene sets suggests that the observed increase of cell invasiveness induced by Ror2 could be driven *via* activation of signaling pathways such as *regulation of actin cytoskeleton* (46), *chemokine signaling pathway* (47, 48), and *ECM receptor interaction* (49). Furthermore, the detection of multiple metabolic pathways supports the evidence of a regulatory connection between the non-canonical Wnt signaling and cancer cell metabolism (50, 51). Although the *Wnt signaling pathway* was not found enriched, this KEGG gene set does not differentiate between the canonical and non-canonical Wnt branches and is therefore less specific. In contrast, the *Calcium signaling pathway* that shares functional overlap with the β-catenin-independent Wnt signaling (52) was identified as significantly enriched in the upregulated as well as all target lists which also points toward the activation of non-canonical Wnt signaling.

As the results of enrichment and Western Blot analyses indicate an induction of the non-canonical Wnt pathway, we further utilized the previously constructed non-canonical Wnt signaling network model (18) for integration of Ror2 targets. We have chosen an approach of a direct projection of the targets onto the signaling network nodes combined with Steiner tree analysis. The identified differentially regulated subnetwork can be considered as non-canonical Wnt module responsive to the signals channeled *via* Ror2 receptor. Into this Ror2/Wnt module Steiner nodes were introduced, which do not embody expression-responsive targets; however, they represent important connector genes that play a central role in network (53). When we focused on the Steiner nodes within the module, we found several of these genes to have already been investigated in the context of aggressive breast cancer, such as *CD36* (54), *CSNK1D* (55), *WNT5A* along with *DVL2* (56), and *PPARGC1A* (57).

In summary, this Ror2/Wnt module highlights the importance of non-canonical Wnt signaling, and in addition to the Ror2 targets it reveals further key genes relevant for breast cancer progression. Furthermore, we were interested whether this pathway module is indeed associated with the observed increased invasiveness of the breast cancer cells. To explore this association in a clinical context, we applied Ror2/Wnt module genes as a prognostic signature in the MFS analysis of a patient cohort.

To this end, the expression profiles of patients were collected across 10 public datasets and metastasis event and follow-up annotations were compiled creating one large compendium dataset. The breast cancer molecular subtypes (LumA, LumB, Her2, and Basal) were predicted within this cohort using the PAM50 signature. However, the predicted classification based on gene signatures should be regarded with caution (58). Therefore, to check reliability of this stratification at a basic biological level, we investigated the four predicted subtype groups in the MFS analysis. The results are consistent with the relapse-free survival observed in the study of Parker et al. (1), which confirms the association of breast cancer subtypes with different metastatic potentials. Along these lines, regarding the 5-year MFS the LumA subtype showed the best prognosis, as expected (59), whereas the Her2 subtype was found to show the worst prognosis, followed by the Basal subtype patients.

The Ror2/Wnt module-based gene signature used for clustering analysis of patient expression profiles revealed four patient subgroups with varying prognosis. The patient cluster with the worst prognosis contains a major proportion of the Basal subtype patients, which is consistent with an increased likelihood of metastasis development in triple-negative breast cancers (60). Also the study of Smid et al. (5) suggested that different activation states of Wnt signaling are associated with different molecular subtypes. However, each subtype was distributed across two or more clusters, which indicates that these clusters do not simply mirror the biology underlying the breast cancer molecular subtypes. Therefore, we applied the Ror2/Wnt module-based gene signature within the individual subtypes to explore whether the subtype-specific differences in metastasis development may be associated with varying expression levels of the module genes.

In both LumA and Basal subtypes we found two patient subgroups significantly differing in MFS. Within the Basal subtype, multiple subgroups have been previously identified and linked to remarkable biological differences (61, 62). Here, we further demonstrated that the expression of Ror2/Wnt module genes has prognostic power in this breast cancer subtype. The luminal patients in contrast to the aggressive subtypes are characterized by continuous relapses occurring in later years (63), which are reflected in the proximity of the two KM curves of the LumA subgroups within the initial years. In contrast to the LumA and Basal subtypes, in the LumB subtype the expression patterns of the module genes are not associated with metastasis development.

Although these results demonstrated the prognostic potential of the Ror2/Wnt module gene signature, the study of Venet et al. (64) has suggested that also random gene signatures are able to separate breast cancer patient into groups with significantly different outcomes. Therefore, to ascertain clinical relevance of the results we compared the performance of the original Ror2/Wnt module gene signature to the randomly generated signatures of the same size sampled from the pool of KEGG database genes. The original signature showed to be more strongly associated with metastasis outcome than the median random signature in the whole cohort as well as in the LumA and Basal subgroups. While in the whole cohort the original signature ranked between the top 9% of the random signatures, in the LumA and Basal subtypes it was 30.5 and 42.9% of the random signatures which showed to be more related to metastasis-free survival than the original, respectively. Therefore, in the view of subtype-specific results the actual clinical utility of the Ror2/Wnt module-based signature seems rather limited. Nevertheless, the analysis of patient data showed an association of the signature with metastasis development complementary to the results of the invasive assays, thus providing a relevant insight into non-canonical Ror2/Wnt signaling.

In conclusion, in this study we explored effects of Wnt5a and Ror2 perturbations in the ER-positive breast cancer cell line MCF-7 on the phenotypic and gene expression levels. We demonstrated that the overexpression of the Ror2 receptor as well as the stimulation with Wnt5a and the combination of both perturbations enhance cancer cell invasion. The expression-responsive targets of Ror2 induce a module of the non-canonical Wnt signaling pathway. Furthermore, these targets alter regulation of further pathways involved in cell remodeling processing and cell metabolism. Moreover, we showed in the gene expression data of breast cancer patients that the Ror2/Wnt module-based gene signature is associated with metastasis-free survival. In summary, these results indicate an important role of non-canonical Wnt signaling mediated *via* the Ror2 receptor in breast cancer progression.

## Author Contributions

MB performed the bioinformatic analyses and wrote the manuscript. KM, FK, and AB designed and performed the wet lab experiments. AW was involved in the RNA-Seq data preprocessing. TP, CB, and AB contributed with clinical expertise. FK, TB, and AB co-conceived and oversaw the study. All authors read and approved the final manuscript.

## Conflict of Interest Statement

The authors declare that the research was conducted in the absence of any commercial or financial relationships that could be construed as a potential conflict of interest. The reviewer, WR, and the handling editor declared their shared affiliation, and the handling editor states that the process nevertheless met the standards of a fair and objective review.

## Abbreviations

Basal, basal-like; DEGs, differentially expressed genes; ER+, estrogen receptor positive; FDR, false-discovery rate; Her2, ERBB2-overexpressing; KM, Kaplan–Meier; LumA, luminal A; LumB, luminal B; MFS, metastasis-free survival.
