# Supplementary figures and images for: Ror2 Signaling and Its Relevance in Breast Cancer Progression
Source: Front Oncol. 2017 Jun 26;7:135. doi: 10.3389/fonc.2017.00135 (PMC5483589; doi:10.3389/fonc.2017.00135)

**norm. protein expression**  
**[% pcDNA]**

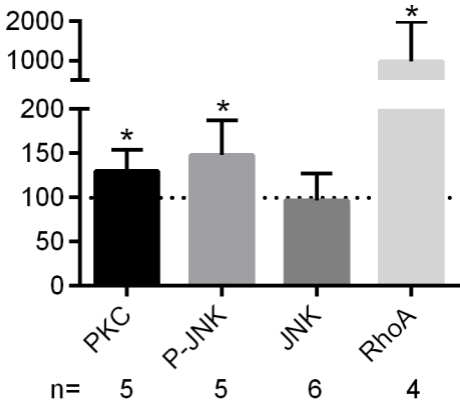

Supplement: Supplementary file 5 [file Image_1.PDF]

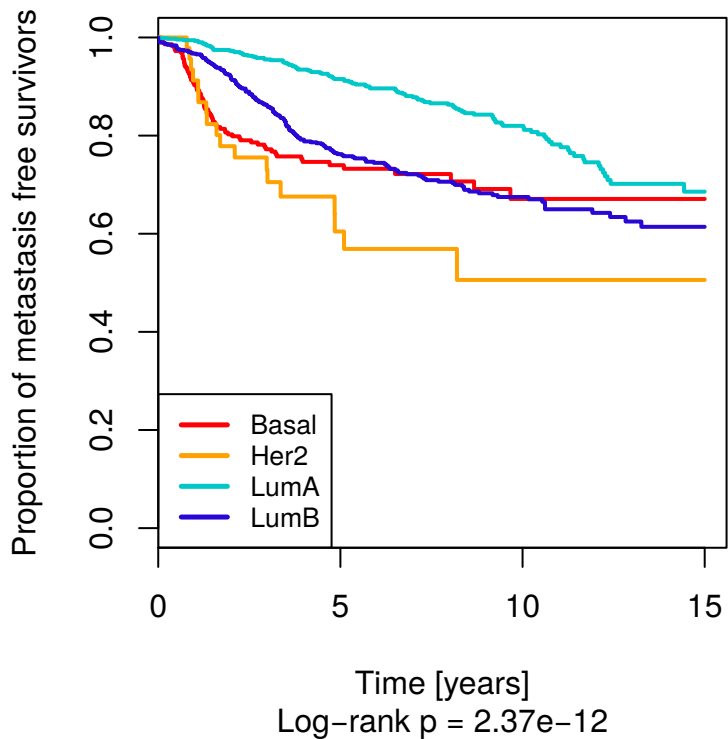

Supplement: Supplementary file 6 [file Image_2.PDF]

# Luminal A

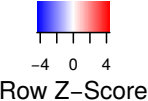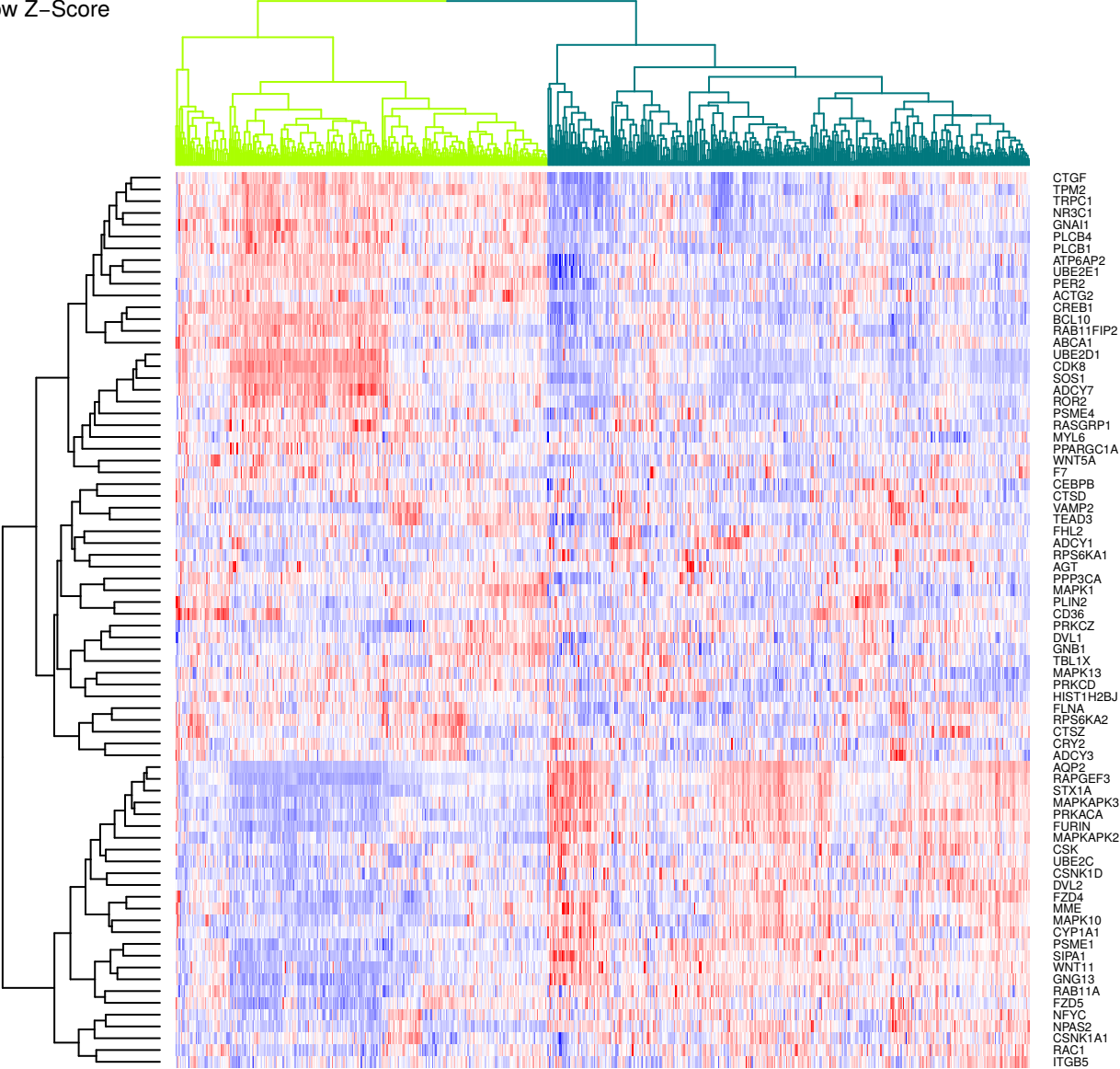

Supplement: Supplementary file 7 [file Image_3.PDF]

Luminal B

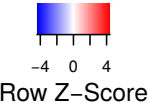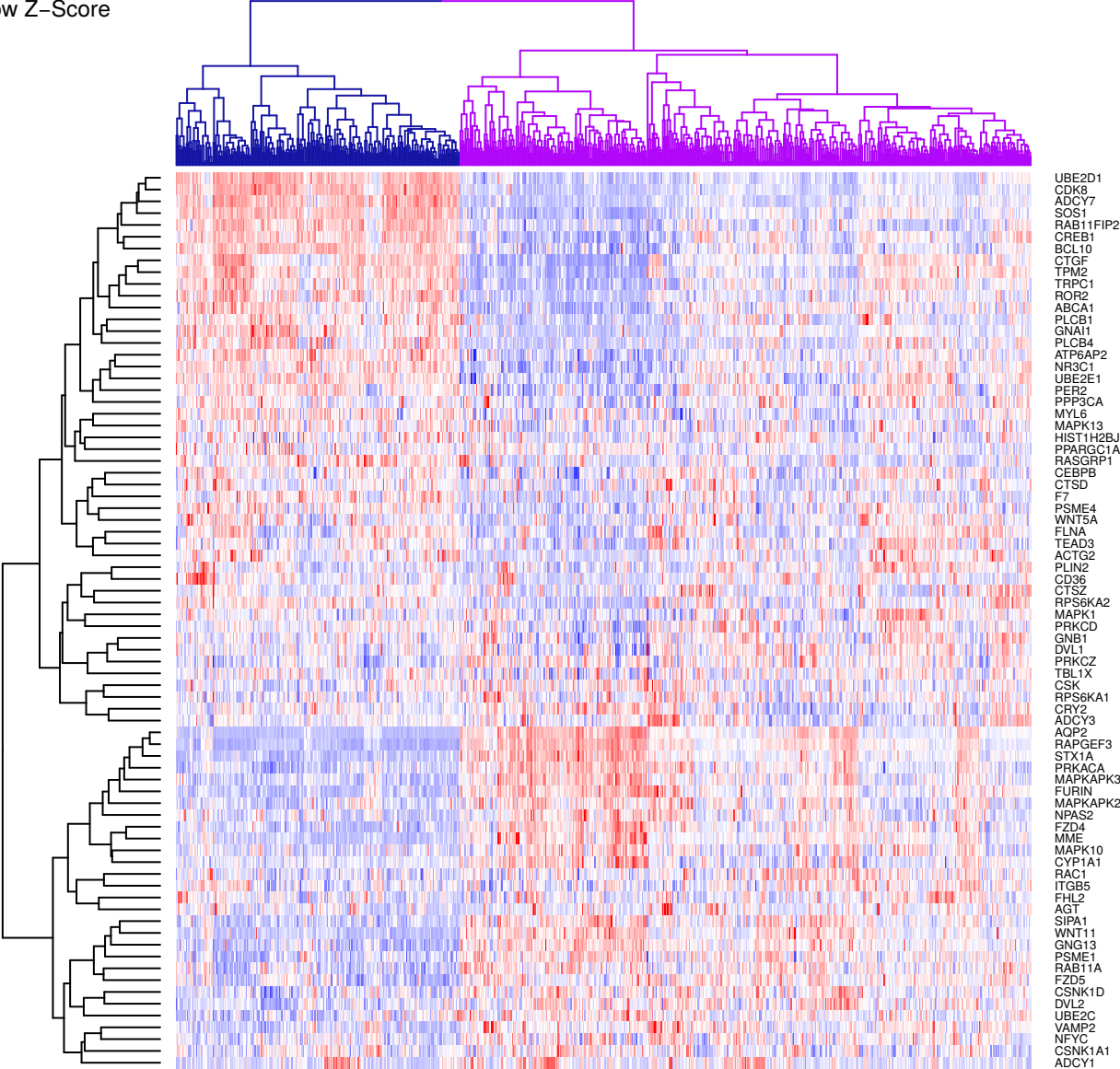

Supplement: Supplementary file 8 [file Image_4.PDF]

# Basal-like

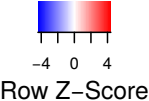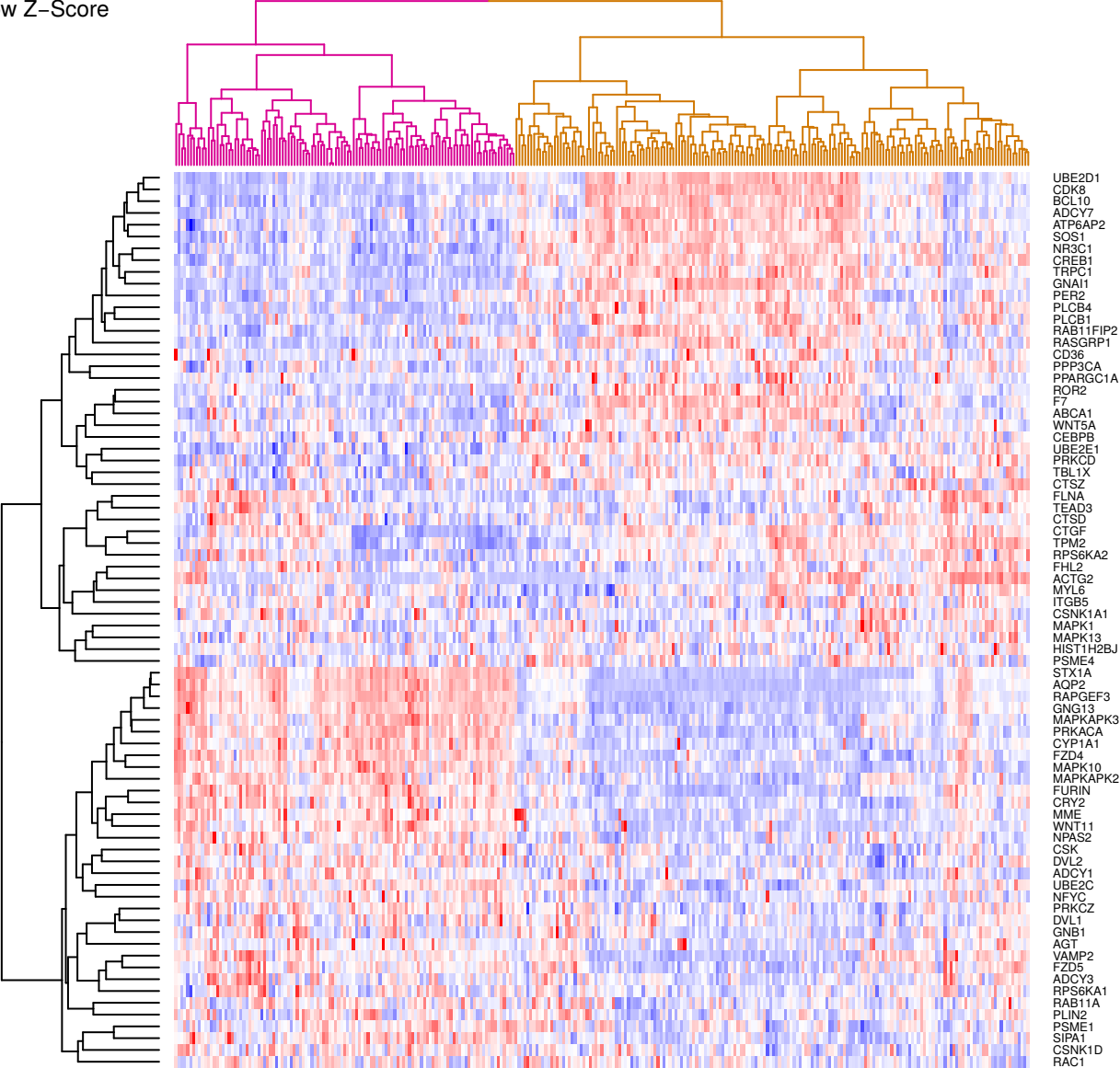

Supplement: Supplementary file 9 [file Image_5.PDF]
